# Supplementary material for: γδ T cells control murine skin inflammation and subcutaneous adipose wasting during chronic Trypanosoma brucei infection
Source: Nat Commun. 2023 Aug 29;14:5279. doi: 10.1038/s41467-023-40962-y (PMC10465518; doi:10.1038/s41467-023-40962-y)
Supplement: Supplementary file 1 — Supplementary Information [file 41467_2023_40962_MOESM1_ESM.pdf]

Supplementary Figures

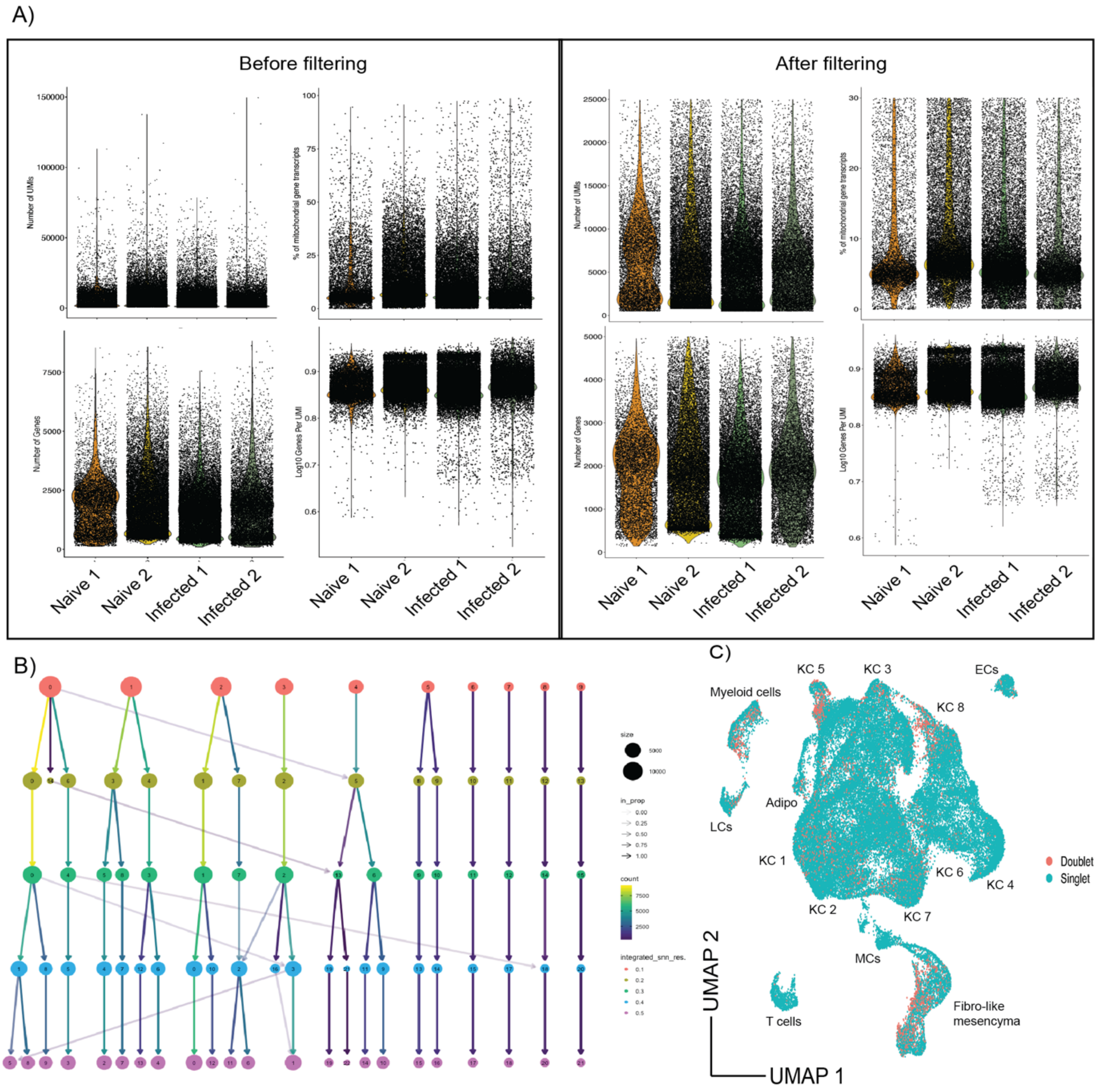

Supplementary figure 1. Quality control measurements of the murine single cell transcriptomics dataset. A) Number of Unique molecular identifies (UMIs), genes, mitochondrial reads, and library complexity (Log10 UMIs/gene) before (left panel) and after (right panel) applying filtering parameters. **B)** Clustree output representing the relationship between different cell clusters at various levels of resolution using the function *FindClusters*. **C)** Dimensionality plot depicting the number of predicted singlets and doublets using DoubletFinder. KC: keratinocytes, FB: fibroblasts, EC: endothelial cells, MCs: Macrophages, LC: Langerhans cells, Adipo: adipocytes, Erythro: erythrocytes.

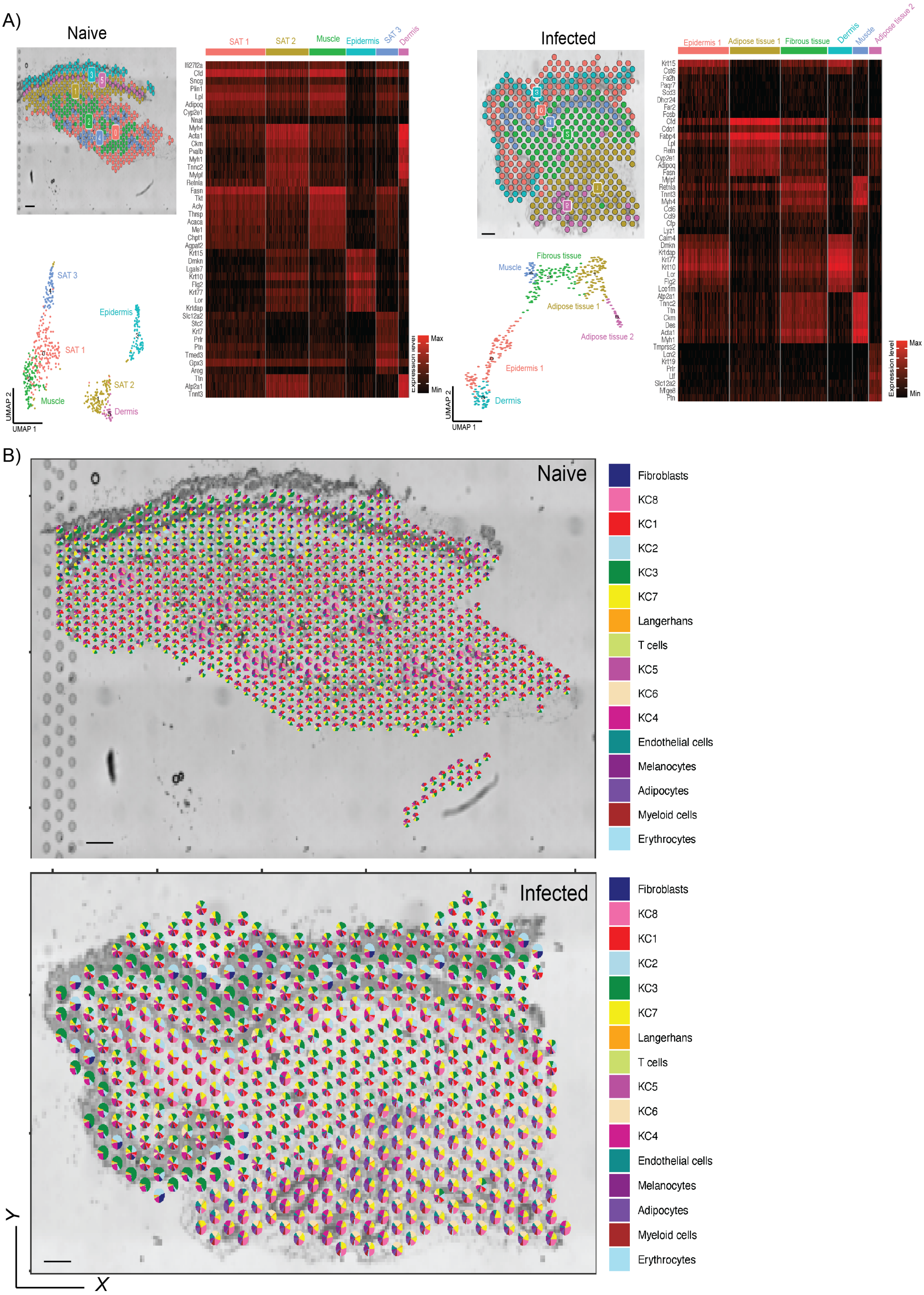
A)
Naive
SAT 1
SAT 2
Muscle
Epidermis
SAT 3
Dermis
UMAP 1
UMAP 2
Expression level
Max
Min
Infected
Epidermis 1
Adipose tissue 1
Fibrous tissue
Adipose tissue 2
Epidermis 1
Dermis
B)
Naive
Infected
Fibroblasts
KC8
KC1
KC2
KC3
KC7
Langerhans
T cells
KC5
KC6
KC4
Endothelial cells
Melanocytes
Adipocytes
Myeloid cells
Erythrocytes
Y
X

Supplementary figure 2. Quality control of 10X Visium datasets from the mouse skin over the course of infection with *T. brucei*. A) Spatial clusters and marker genes for each spatial cluster in the naïve (**left panel**) and infected (**right panel**) murine skin using 10X Visium spatial transcriptomics. **B)** Spatial transcriptomics spot deconvolution analysis for the naïve (top) and infected (bottom) skin sections using the Giotto package. The spot deconvolution was conducted using the single cell data in Figure 1C. Scale bar, 100 μm. KC: keratinocytes, FB: fibroblasts, EC: endothelial cells, MCs: Macrophages, LC: Langerhans cells, Adipo: adipocytes, Erythro: erythrocytes.

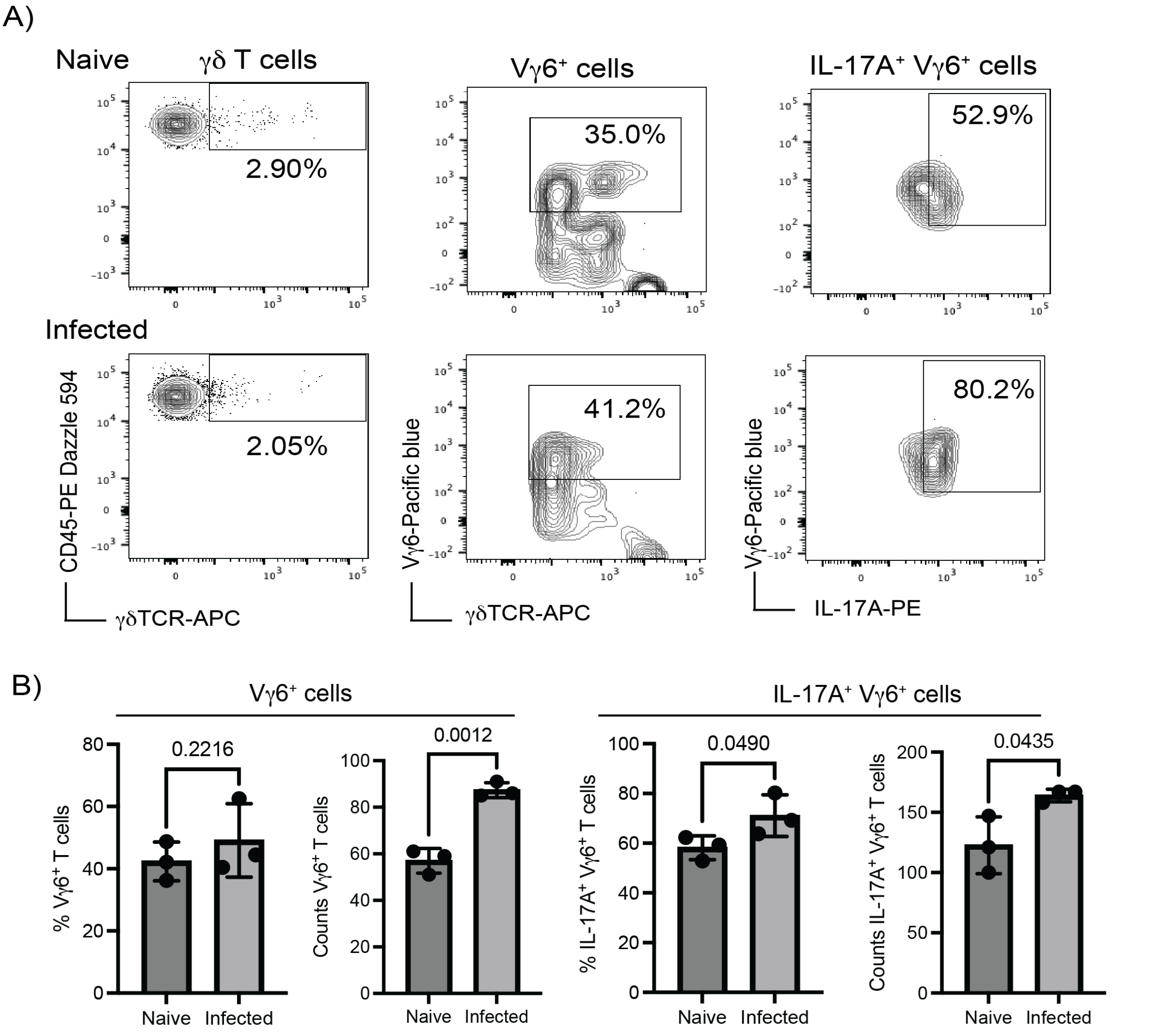

Supplementary figure 3. Chronic *T. brucei* infection induces an expansion of IL-17⁺ Vγ6⁺ cells in the murine skin. A) Representative flow cytometry data showing the frequencies of γδ T cells, Vγ6⁺ cells, and IL-17⁺ Vγ6⁺ cells in naïve and infected skin sections. **B)** Quantification of Vγ6⁺ cells and IL-17⁺ Vγ6⁺ cells as measured by flow cytometry (n = 3 mice/group). *T* test comparison between infected samples. A *p* value <0.05 is considered significant. Source data are provided as a Source data file.

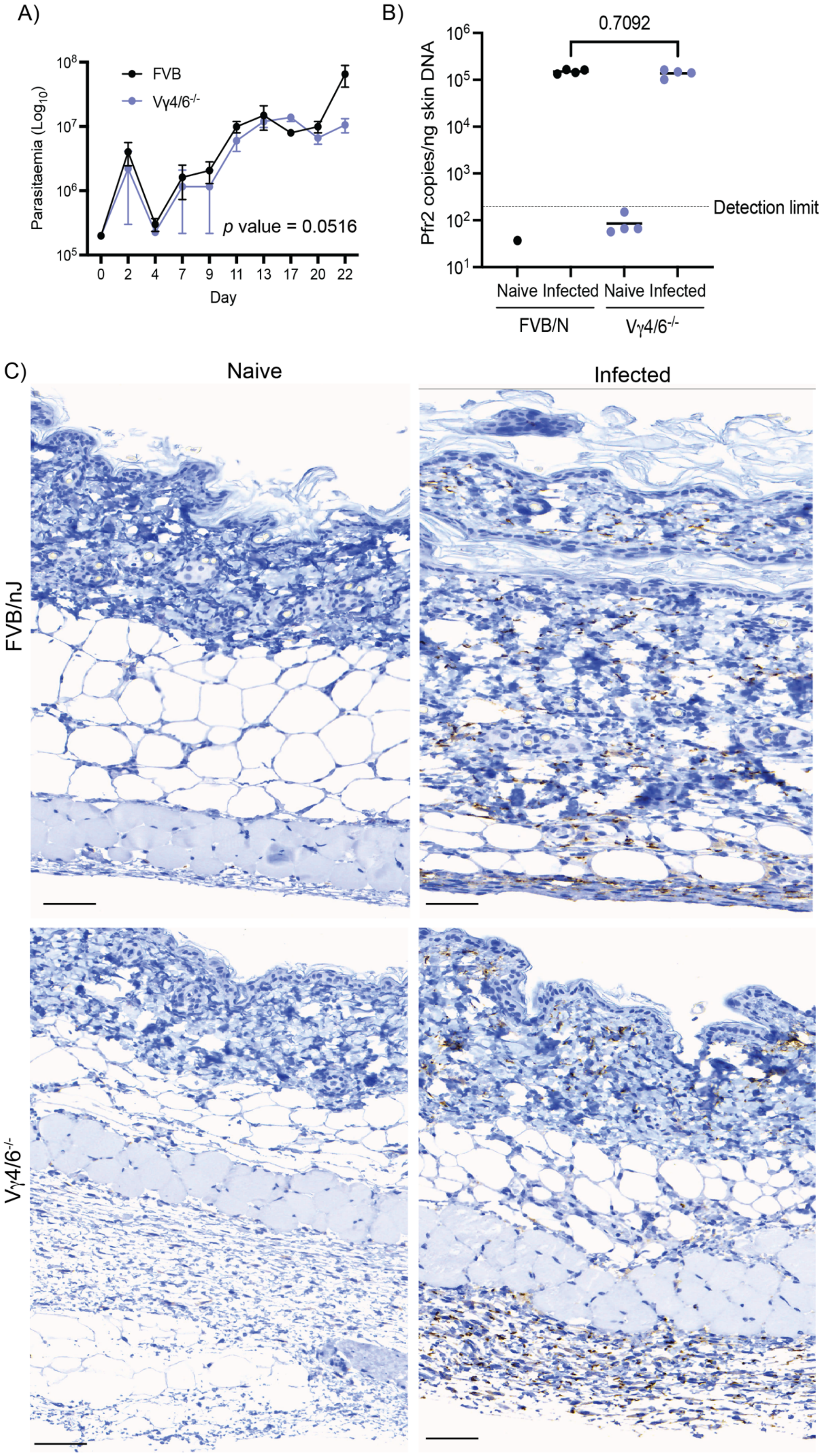
A)
Parasitaemia (Log10)
FVB
Vγ4/6-/-
p value = 0.0516
Day
B)
Pfr2 copies/ng skin DNA
0.7092
Detection limit
Naive Infected Naive Infected
FVB/N
Vγ4/6-/-
C)
Naive
Infected
FVB/nJ
Vγ4/6-/-

Supplementary figure 4. Characterisation of parasites in the skin of Vγ4/6$^{-/-}$ mice during *T. brucei* infection. A) Measurement of circulating parasitaemia in female FVB/N (n = 4) and Vγ4/6$^{-/-}$ mice (n = 4) over a period of 22 days. One-way ANOVA test with multiple corrections. A p value <0.05 is considered significant. Source data are provided as a Source data file. **B)** qRT-PCR of skin-dwelling trypanosomes, measuring the trypanosome-specific gene *Prf2*. *Prf2* copy numbers were normalised per ng of skin DNA from naïve and infected FVB/N and Vγ4/6$^{-/-}$ mice (n = 4 mice/group). *T* test comparison between infected samples. A p value <0.05 is considered significant. The detection limit, as measured in skin biopsies from naïve controls, is also indicated with a dotted line. Source data are provided as a Source data file. **C)** Representative immunohistochemistry of murine skin biopsies from naïve and infected FVB/N and Vγ4/6$^{-/-}$ mice to detect the presence of *T. brucei* using an antibody against the trypanosome-specific luminal binding protein 1 (BiP). Scale bar = 50 μm.

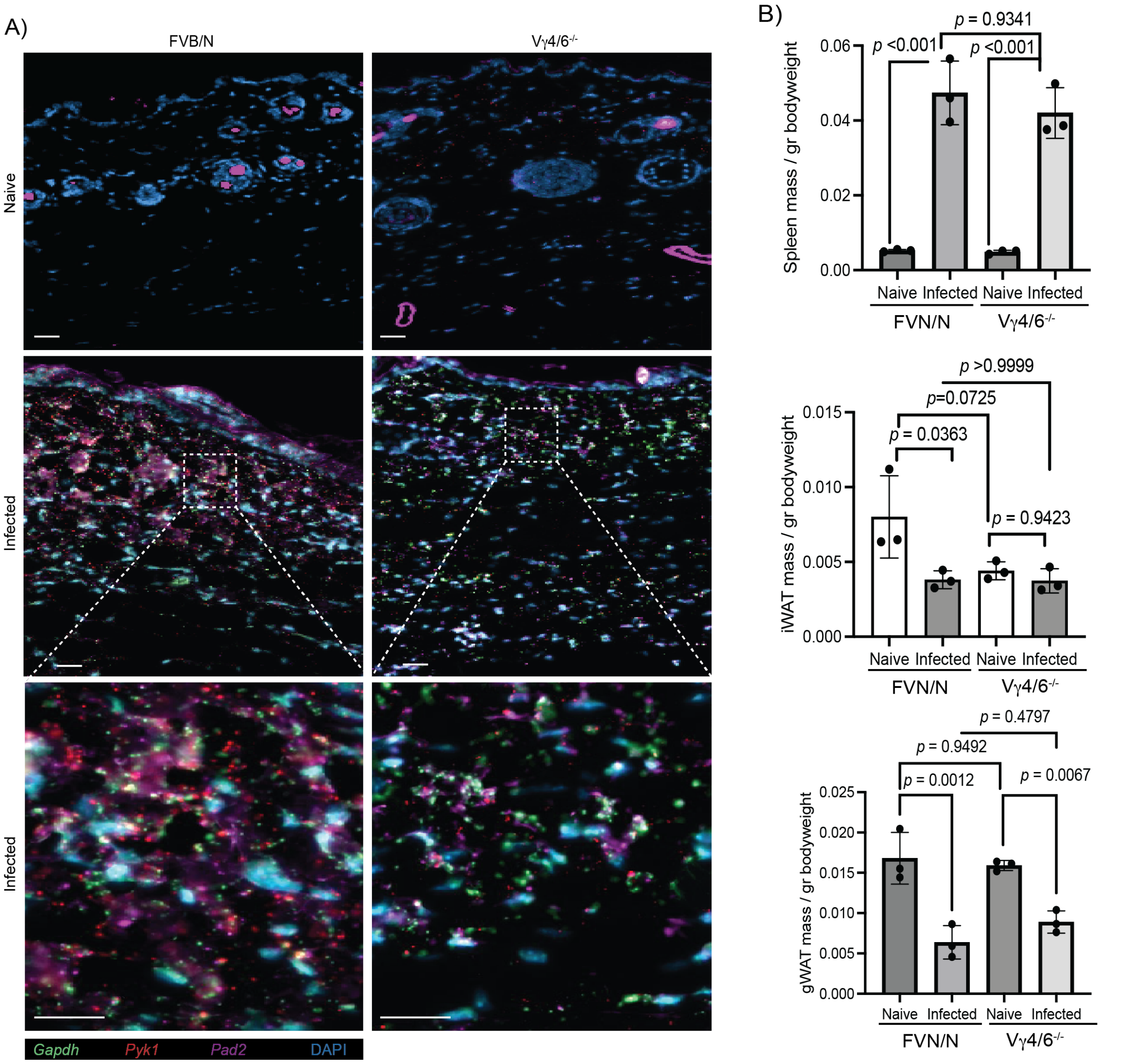

Supplementary figure 5. Single molecule fluorescent *in situ* hybridisation (smFISH) analysis showing the spatial distribution of *T. brucei* developmental stages in the skin of FVB/N and Vγ4/6$^{-/-}$ mice. A) Top panels: skin sections from naïve FVB/N and Vγ4/6$^{-/-}$ mice. **Middle panels**: skin sections from infected FVB/N and Vγ4/6$^{-/-}$ mice depicting the expression of the *T. brucei*-specific transcripts *Gapdh* and *Pyk1* (slender specific markers) and *Pad2* (stumpy specific marker). The dotted square indicates an area selected for magnification. **Bottom panels:** Magnified fields of the infected samples showing the distribution of both slender and stumpy markers in the skin of FVB/N and Vγ4/6$^{-/-}$ mice. Scale bar: 50 μm. **B)** Spleen (top), subcutaneous white adipose tissue (middle), and gonadal white adipose tissue weight (bottom), normalised to bodyweight, from naïve and infected FVB/N and Vγ4/6$^{-/-}$ mice (n = 3

mice/group). One-way ANOVA test with multiple corrections. A p value <0.05 is considered significant. Source data are provided as a Source data file.

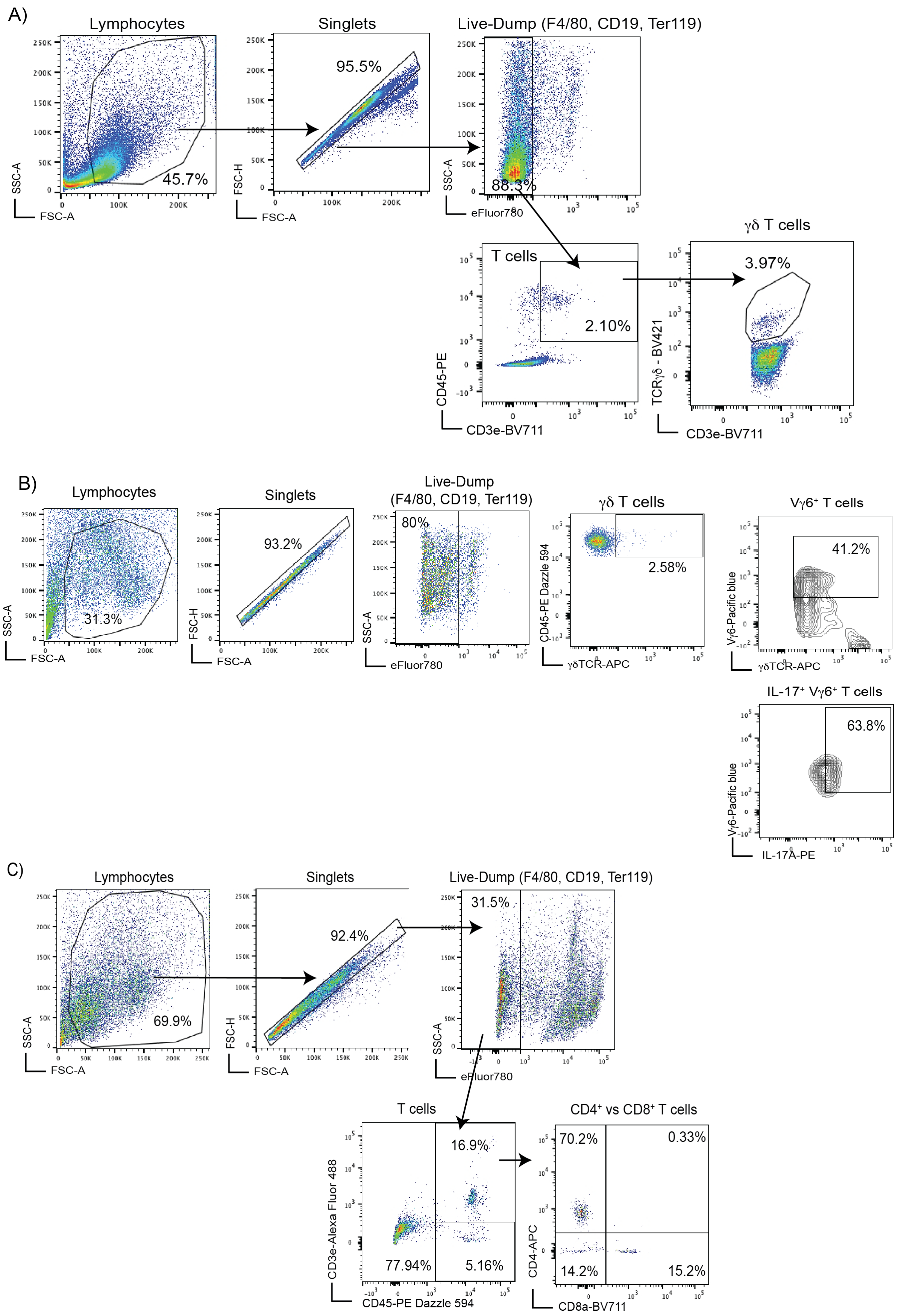
A)
Lymphocytes
45.7%
SSC-A
FSC-A
Singlets
95.5%
FSC-H
FSC-A
Live-Dump (F4/80, CD19, Ter119)
88.3%
SSC-A
eFluor780
T cells
2.10%
CD45-PE
CD3e-BV711
γδ T cells
3.97%
TCRγδ - BV421
CD3e-BV711
B)
Lymphocytes
31.3%
SSC-A
FSC-A
Singlets
93.2%
FSC-H
FSC-A
Live-Dump
(F4/80, CD19, Ter119)
80%
SSC-A
eFluor780
γδ T cells
2.58%
CD45-PE Dazzle 594
γδTCR-APC
Vγ6⁺ T cells
41.2%
Vγ6-Pacific blue
γδTCR-APC
IL-17⁺ Vγ6⁺ T cells
63.8%
Vγ6-Pacific blue
IL-17A-PE
C)
Lymphocytes
69.9%
SSC-A
FSC-A
Singlets
92.4%
FSC-H
FSC-A
Live-Dump (F4/80, CD19, Ter119)
31.5%
SSC-A
eFluor780
T cells
16.9%
77.94%
5.16%
CD3e-Alexa Fluor 488
CD45-PE Dazzle 594
CD4⁺ vs CD8⁺ T cells
70.2%
0.33%
14.2%
15.2%
CD4-APC
CD8a-BV711

Supplementary figure 6. Flow cytometry analysis of skin-resident T lymphocytes. Gating strategy for the identification of skin γδ T cells (**A**), IL-17$^+$ Vγ6$^+$ cells (B), and INFγ-producing CD4$^+$ and CD8$^+$ T cells (**C**).

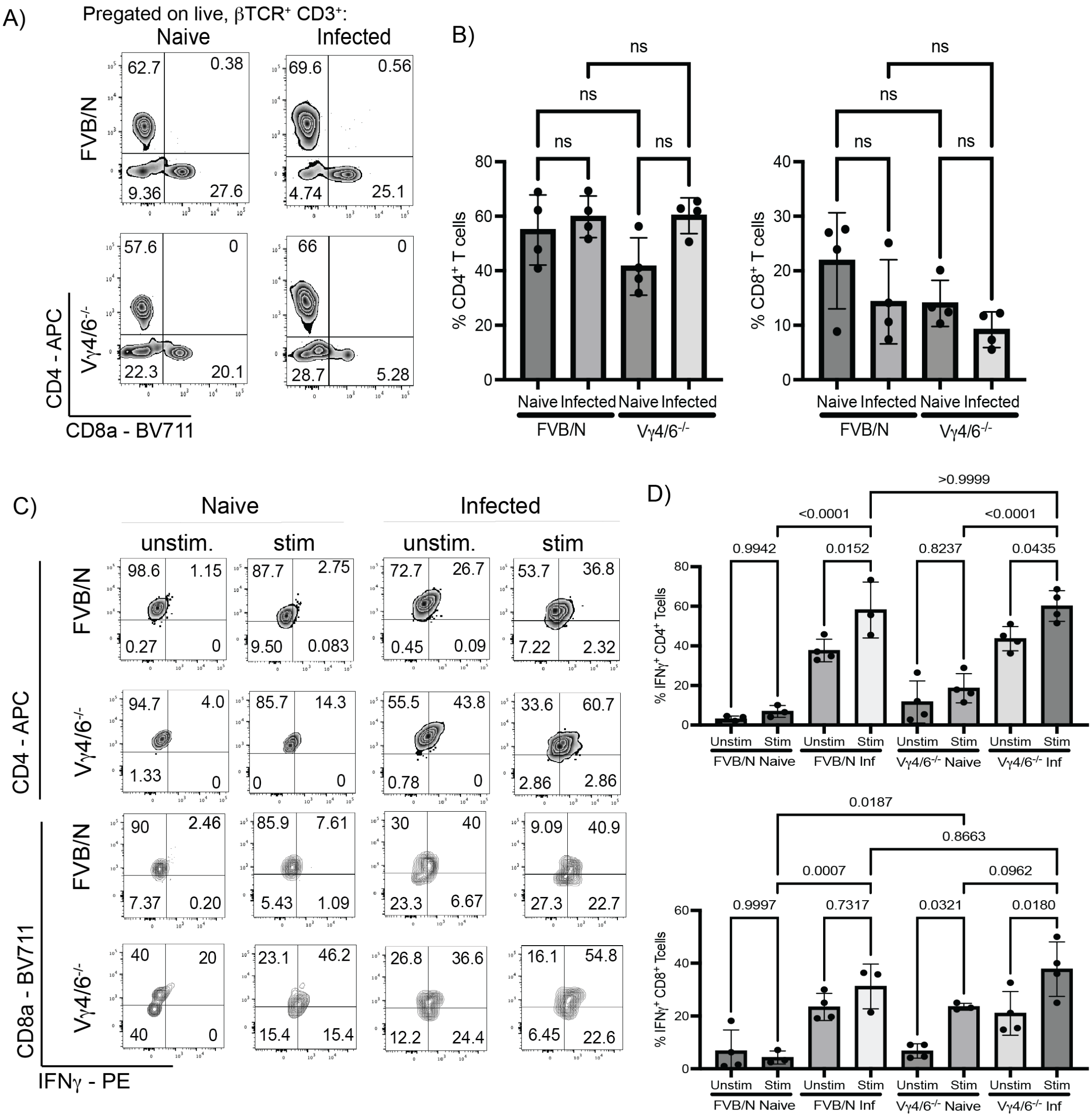

Supplementary figure 7. Quantification of skin-resident lymphocytes in the Vγ4/6$^{-/-}$ mice during *T. brucei* infection. A) Representative flow cytometry analysis of skin CD4$^+$ and CD8$^+$ T cells in naïve and infected FVB/N and Vγ4/6$^{-/-}$ mice (*n* = 4 mice/group). **B)** Quantification of the frequency of skin CD4$^+$ (left panel) and CD8$^+$ T cells (right panel) in naïve and infected FVB/N and Vγ4/6$^{-/-}$ mice (*n* = 4 mice/group) as shown in (C). ANOVA test with multiple corrections. A *p* value <0.05 is considered significant. Source data are provided as a Source data file. **C)** Representative flow cytometry analysis of *ex vivo* recall assay to determine the production of IFNγ in skin-resident CD4$^+$ and CD8$^+$ T cells in naïve and infected FVB/N and Vγ4/6$^{-/-}$ mice (*n* = 4 mice/group). **D)** Quantification of the frequency of skin CD4$^+$ (top panel) and CD8$^+$ T

cells (bottom panel) in naïve and infected FVB/N and Vγ4/6$^{-/-}$ mice (*n* = 4 mice/group) as shown in (C). Source data are provided as a Source data file.
