# Supplementary material for: γδ T cells control murine skin inflammation and subcutaneous adipose wasting during chronic Trypanosoma brucei infection
Source: Nat Commun. 2023 Aug 29;14:5279. doi: 10.1038/s41467-023-40962-y (PMC10465518; doi:10.1038/s41467-023-40962-y)
Supplement: Supplementary file 3 — Description of Additional Supplementary Files [file 41467_2023_40962_MOESM3_ESM.pdf]

Supplementary Data legend

Supplementary Data 1. Overview of the mouse skin single cell transcriptomics during chronic *T. brucei* infection. S1A) Quality control including mean reads per cell and median genes per cell before and after filtering out low quality cell types. **S1B)** Overview of the major cell types detected in the single cell dataset at a resolution of 0.4. The marker genes are also included. **S1C)** Overview of the stromal cells detected in the skin dataset at a resolution of 0.3. The marker genes for these clusters, as well as representative UMAP plots are also included. **S1D)** As in S1C, but for the myeloid cells at a resolution of 0.3**. S1E)** As in S1C, but for the T cells at a resolution of 0.3**.**

Supplementary Data 2. Overview of the spatial transcriptomics of the mouse skin during chronic *T. brucei* infection. S2A) Overview of the spatial transcriptomics project, including total number of reads sequenced per biological replicate, the median number of genes per spot and the percentage of mappable reads to the mouse genome (mm10). **S2B)** Mouse marker genes identified in the 10X Visium spatial transcriptomics datasets.

Supplementary Data 3. Histopathological analysis of biopsies taken from naïve and infected FVB/NJ and Vγ4/6$^{-/-}$ mice. Skin biopsies were harvested at 21 days post-infection (*n* = 4 mice/group), fixed in 10% PFA and counterstained with the *T. brucei*-specific antibody TbBiP. Uninfected animals (*n* = 4) were included as naïve controls. Histological examinations were scored using H&E staining and BiP staining and was conducted double-blinded. The results reported in this table are in comparison to naïve controls for the corresponding genetic background, and encompass a detailed analysis of the epidermis, dermis, hypodermis, skeletal muscle, and the subcutaneous adipose tissue. The column labelled "BiP" (columns AD to AI) represents the results from the immunohistochemistry analysis against the anti-Trypanosoma anti-BiP antibody.
