# Supplementary material for: γδ T cells control murine skin inflammation and subcutaneous adipose wasting during chronic Trypanosoma brucei infection
Source: Nat Commun. 2023 Aug 29;14:5279. doi: 10.1038/s41467-023-40962-y (PMC10465518; doi:10.1038/s41467-023-40962-y)
Supplement: Supplementary file 7 — Reporting Summary [file 41467_2023_40962_MOESM7_ESM.pdf]

nature portfolio

Corresponding author(s): Juan F. Quintana

Last updated by author(s): 22/06/2023

Reporting Summary

Nature Portfolio wishes to improve the reproducibility of the work that we publish. This form provides structure for consistency and transparency in reporting. For further information on Nature Portfolio policies, see our Editorial Policies and the Editorial Policy Checklist.

Statistics

For all statistical analyses, confirm that the following items are present in the figure legend, table legend, main text, or Methods section.

n/a	Confirmed	
☐	☒	The exact sample size (*n*) for each experimental group/condition, given as a discrete number and unit of measurement
☐	☒	A statement on whether measurements were taken from distinct samples or whether the same sample was measured repeatedly
☐	☒	The statistical test(s) used AND whether they are one- or two-sided<br>*Only common tests should be described solely by name; describe more complex techniques in the Methods section.*
☒	☐	A description of all covariates tested
☐	☒	A description of any assumptions or corrections, such as tests of normality and adjustment for multiple comparisons
☐	☒	A full description of the statistical parameters including central tendency (e.g. means) or other basic estimates (e.g. regression coefficient) AND variation (e.g. standard deviation) or associated estimates of uncertainty (e.g. confidence intervals)
☐	☒	For null hypothesis testing, the test statistic (e.g. *F*, *t*, *r*) with confidence intervals, effect sizes, degrees of freedom and *P* value noted<br>*Give P values as exact values whenever suitable.*
☒	☐	For Bayesian analysis, information on the choice of priors and Markov chain Monte Carlo settings
☒	☐	For hierarchical and complex designs, identification of the appropriate level for tests and full reporting of outcomes
☒	☐	Estimates of effect sizes (e.g. Cohen's *d*, Pearson's *r*), indicating how they were calculated

Our web collection on statistics for biologists contains articles on many of the points above.

Software and code

Policy information about availability of computer code

Data collection	Single cell RNA sequencing was conducted on an Illumina Novaseq 6000 sequencers by Glasgow polyomics. Spatial RNA sequencing was conducted on a NextSeq 550 Illumina instrument at GenomeScan. Fastq sequence files were de-multiplexed, aligned, and annotated using a reference combined mouse (mmu10; https://www.ncbi.nlm.nih.gov/assembly/GCF_000001635.20/) and and Cell Ranger software (vCR6.1; single cell RNA sequencing) or Space Ranger (vSR1.3; spatial RNA sequencing) softwares. Gene expression was counted using unique molecular identifier barcodes, and gene-cell matrices were constructed. FACS DIVA software (v9.0) was used for acquisition of flow cytometry data.
Data analysis	The following R packages were used to analyse the single cell and spatial sequencing dataset: R (v4.2.1), Seurat (v4.1.0), sctransform (v0.3.3), RcolorBrewer (v1.1.2), nichenetr (v1.1.0). Fiji v2 was used for image analysis. FlowJo (v10.8.2) (BD) were used for analysis of flow cytometry data. Code used to perform analysis described can be accessed at Zenodo (DOI: 10.5281/zenodo.7677469).

For manuscripts utilizing custom algorithms or software that are central to the research but not yet described in published literature, software must be made available to editors and reviewers. We strongly encourage code deposition in a community repository (e.g. GitHub). See the Nature Portfolio guidelines for submitting code & software for further information.

Data

Policy information about availability of data

All manuscripts must include a data availability statement. This statement should provide the following information, where applicable:

- Accession codes, unique identifiers, or web links for publicly available datasets
- A description of any restrictions on data availability
- For clinical datasets or third party data, please ensure that the statement adheres to our policy

The data generated in this study have been deposited in the Gene Expression Omnibus database under accession code GSE226113 (https://www.ncbi.nlm.nih.gov/geo/query/acc.cgi?&acc=GSE226113). A reviewer's token in included here: mlmroswwnraxbwn. The processed transcript count data and cell metadata generated in this study are available at Zenodo (DOI: 10.5281/zenodo.7677469). The flow cytometry data generated in this study are provided in the Supplementary Information/Source Data file.

Human research participants

Policy information about studies involving human research participants and Sex and Gender in Research.

Reporting on sex and gender	No human samples or subjects were used in this study
Population characteristics	No human samples or subjects were used in this study
Recruitment	No human samples or subjects were used in this study
Ethics oversight	No human samples or subjects were used in this study

Note that full information on the approval of the study protocol must also be provided in the manuscript.

Field-specific reporting

Please select the one below that is the best fit for your research. If you are not sure, read the appropriate sections before making your selection.

☒ Life sciences ☐ Behavioural & social sciences ☐ Ecological, evolutionary & environmental sciences

For a reference copy of the document with all sections, see nature.com/documents/nr-reporting-summary-flat.pdf

Life sciences study design

All studies must disclose on these points even when the disclosure is negative.

Sample size	The maximum sample size was used for all the transcriptomics experiments presented in this study, within the limitations of the technology implemented (~10,000 cells per experiment, and 4 tissue sections per spatial transcriptomic slide). For in vivo experiments, we used a sample size of 4-5 animals per experimental condition. This sample size is based on measures of variance from historical data in the laboratory, and is enough to detect differences between experimental and control groups at a 5% significance level and 90% power.
Data exclusions	No data were excluded from the analysis
Replication	All experiments were conducted using independent biological replicates (at least n = 2 for single cell transcriptomics). The data obtained from the single cell experiments were succesfully replicated in independent in vivo experiments (e.g., expansion of CD27- gdT cells in the skin of female BALB/c mice). Spatial transcriptomics datasets were not replicated due to costs and logistics, but were independently validated using in situ hybridisation approaches. All flow cytometry measurements were conducted in at least two independent experiments with similar results.
Randomization	Randomisation of scRNAseq or spatial transcriptomics was not appropriate as both naive and infected animals were processed in parallel and required additional labelling for downstream analysis (e.g., flow cytometry analysis, H&E analysis). Randomisation of in vivo experiments was not appropriate as the infected animals required to be closely monitored for the development of clinical adverse effects. Animals used for flow cytometry analysis described here as validation for single cell and spatial transcriptomics were randomly allocated into cages.
Blinding	Blinding of scRNA was not appropriate as data processing and analysis need to be carried out appropriately and differentially for biological between experimental conditions. Measurements associated with parasitaemia and clinical scoring were conducted by a trained scientist blinded to the study. For flow cytometry analysis, blinding was not carried out in order to allow inclusion of appropriate measurements of background intensity and signal adjustments during acquisition.

Reporting for specific materials, systems and methods

We require information from authors about some types of materials, experimental systems and methods used in many studies. Here, indicate whether each material, system or method listed is relevant to your study. If you are not sure if a list item applies to your research, read the appropriate section before selecting a response.

Materials & experimental systems

n/a	Involved in the study
[ ]	[x] Antibodies
[ ]	[x] Eukaryotic cell lines
[x]	[ ] Palaeontology and archaeology
[ ]	[x] Animals and other organisms
[x]	[ ] Clinical data
[x]	[ ] Dual use research of concern

Methods

n/a	Involved in the study
[x]	[ ] ChIP-seq
[ ]	[x] Flow cytometry
[x]	[ ] MRI-based neuroimaging

Antibodies

Antibodies used	The following anti-mouse antibodies were used for flow cytometry experiments: Fixable viability dye eFluor780 (Thermo, 1/1,000), CD45-PE (Biolegend, clone HI30, 1/400), CD45-PE Dazzle 594 (Biolegend, clone 30-F11, 1/400), CD19 PE-Cy7 (Biolegend, clone 1D3/CD19, 1/400), F4/80 PE-Cy7 (Biolegend, clone BM8, 1/400), TER119 PE-Cy7 (Biolegend, clone TER-119, 1/400), CD3e-PE Dazzle 594 (Biolegend, clone KT3.1.1, 1/400), TCRgd Brilliant Violet 421 (Biolegend, clone GL3, 1/400), CD27 APC (Biolegend, clone LG.3A10, 1/400), CD4 APC (Biolegend, clone GK1.5, 1/400), CD8a Brilliant Violet 711 (Biolegend, clone 53-6.7, 1/400), IFNg PE (Biolegend, clone XMG1.2, 1/400), CD3e Alexa Fluor 488 (Biolegend, clone 500A2, 1/400), IL-17A. The Vg6 antibody (Pacific blue; 1:50) was kindly provided by Shinya Hatano and Yasunobu Yoshikai (Kyushu University, Fukuoka, Japan).
Validation	All antibodies for flow cytometry were used at the recommended concentration by the manufacturer and tested in pilot titration experiments. The antibodies used for imaging were used exactly as recommended by the manufacturer, including the concentration.

Eukaryotic cell lines

Policy information about cell lines and Sex and Gender in Research

Cell line source(s)	No cell lines were used in this study
Authentication	Not required
Mycoplasma contamination	Not required
Commonly misidentified lines (See ICLAC register)	Not required

Animals and other research organisms

Policy information about studies involving animals; ARRIVE guidelines recommended for reporting animal research, and Sex and Gender in Research

Laboratory animals	Six-to-eight-week-old wild-type female BALB/c (stock 000651) and FVB/nJ mice (stock 001800) were purchased from Jackson Laboratories. Tcrd—/— mice (a gift from Adrian Hayday, Francis Crick Institute) and Vg4/6—/— mice (a gift from Rebecca O'Brien, National Jewish Health) were backcrossed to FVB/n background N10. Female mice aged (6-8 weeks old) were used for infection. Mice were age and weight-matched within experiments. Animals were housed on a 12 h light-dark cycle and fed ad libitum. Room temperature was between 20-24C and humidity was between 50-70%.
Wild animals	No wild animals were used in this study
Reporting on sex	Only female mice were used in this study. Historical data in the laboratory suggests that both sexes respond similarly to infection by Trypanosoma brucei.
Field-collected samples	No field samples were used in this study
Ethics oversight	All animal experiments were approved by the University of Glasgow Ethical Review Committee and performed in accordance with the home office guidelines, UK Animals (Scientific Procedures) Act, 1986 and EU directive 2010/63/EU. All experiments were conducted under SAPO regulations and UK Home Office project licence number PC8C3B25C to Dr. Jean Rodgers. The in vivo work related to the single cell and spatial transcriptomic experiments were conducted at 21 post-infection (dpi) and correlated with increased clinical scores and procedural severity.

Note that full information on the approval of the study protocol must also be provided in the manuscript.

Flow Cytometry

Plots

Confirm that:

- [x] The axis labels state the marker and fluorochrome used (e.g. CD4-FITC).
- [x] The axis scales are clearly visible. Include numbers along axes only for bottom left plot of group (a 'group' is an analysis of identical markers).
- [x] All plots are contour plots with outliers or pseudocolor plots.
- [x] A numerical value for number of cells or percentage (with statistics) is provided.

Methodology

Sample preparation

Mice were euthanised as described above and transcardially perfused with ice-cold 0.025% (wt/vol) EDTA in 1X PBS. Hair was shaved from the abdominal flank area and skin biopsies were then collected. Some of these sections were placed in 4% PFA for 16 hours prior to embedding in paraffin and histological analysis. For single cell analysis, excised 4cm2 skin sections were minced into small pieces with a scalpel blade and enzymatically digested with Collagenase type I (500 U/ml; Gibco) and DNAse I (1 mg/ml; Sigma) in HBSS containing 0.04% BSA (Invitrogen) for ~1 hr at 37 °C with shaking at 300rpm. Liberated cells from the partially digested tissue were pushed through a 70 um nylon mesh filter with an equal volume of HBSS 0.04% BSA, and then kept on ice. The tissue remaining in the 70 um filter was incubated in 0.05% trypsin EDTA for 15 minutes at 37°C, and then liberated cells pushed through the filter with an equal of HBSS 0.04% BSA. Flowthrough from the two digestion steps were combined, passed through a 40 uM filter to remove any cell aggregate and spun at 350g for 10mins at 4oC. For flow cytometry analysis, single cell suspensions were resuspended in ice-cold FACS buffer (2mM EDTA, 5 U/ml DNAse I, 25mM HEPES and 2.5% Foetal calf serum (FCS) in 1X PBS) and stained for extracellular markers. The list of flow cytometry antibodies used in this study were obtained from Biolegend and are presented in the table below. Samples were run on a flow cytometer LSRFortessa (BD Biosciences) and analysed using FlowJo software version 10 (Treestar). For intracellular staining, single-cell isolates from brain or draining lymph nodes were stimulated as above in Iscove's modified Dulbecco's media (supplemented with 1X non-essential amino acids, 50 U/ml penicillin, 50 ug/ml streptomycin, 50µM β-mercaptoethanol, 1 mM sodium pyruvate and 10%FBS. Gibco). Cells were then permeabilized with a Foxp3/Transcription Factor Staining Buffer Set (eBioscience) and stained overnight at 4°C.

Instrument

BD Fortessa Flow Cytometre (BD Biosciences)

Software

FACS Diva software (v9.0) and FlowJo (vl0.8.2) (BD).

Cell population abundance

25 µ of liquid compensation beads (BD Biosciences, 335925) were added to allow for quantification of absolute cell numbers.

Gating strategy

Unstained controls and isotype controls were used to identify background staining levels and determine gate placement. Doublets were excluded based on linearity of FSC-A and FSC-H. From singlets, live cells were identified as the fixable eFluor 780. T cell lymphocytes were detected by co-expression of CD45 and CD3e, and further subsetted into either gdT cells (TCRgd +), or conventional T cells (TCRgd-). The conventional population was further divided into either CD4 or CD8 T cells. For ex vivo stimulation assays, we focussed on measuring IFNg levels in both CD4+ and CD8+ conventional T cells.

To determine the presence of IL-17A+ producing Vg6+ cells in the skin, we proceeded as above to gate on live cells excluding myeloid cells, B cells, and erythrocytes. We then gated on CD45+ vs gdTCR+ douple positive cells to detect all dermal gd T cells, then on Vg6+ gdTCR+ cells (to detect Vg6+ cells), and then in Vg6+ IL-17+ cells to identify IL-17+ producing Vg6+ cells in the skin.

- [x] Tick this box to confirm that a figure exemplifying the gating strategy is provided in the Supplementary Information.
